# *Leptoria phrygia* in Southern Taiwan shuffles and switches symbionts to resist thermal-induced bleaching

**DOI:** 10.1038/s41598-020-64749-z

**Published:** 2020-05-08

**Authors:** Ya-Yi Huang, Rodrigo Carballo-Bolaños, Chao-Yang Kuo, Shashank Keshavmurthy, Chaolun A. Chen

**Affiliations:** 1 Biodiversity Research Center, Academia Sinica, Taipei, Taiwan; 2 Biodiversity Program, Taiwan International Graduate Program, Academia Sinica; National Taiwan Normal University, Taipei, Taiwan; 3 Institute of Oceanography, National Taiwan University, Taipei, Taiwan; 4 Department of Life Science, Tung-Hai University, Taichung, Taiwan

**Keywords:** Metagenomics, Marine biology, Climate-change ecology

## Abstract

Symbiodiniaceae communities in some corals often shuffle or switch after severe bleaching events, one of the major threats to coral survival in a world with climate change. In this study we reciprocally transplanted five *Leptoria phrygia* colonies between two sites with significantly different temperature regimes and monitored them for 12 months. Our ITS2 amplicon deep sequencing demonstrated that *L*. *phrygia* acclimatized to maintain a strong and stable association with *Durusdinium* D17, *D*. *trenchii*, and *D*. *glynnii*, but also remained flexible and formed a short-term association with different *Cladocopium*. Most interestingly, two colonies shuffled between *Durusdinium* and *Cladocopium* without the occurrence of bleaching; one colony even switched its dominant *Cladocopium* after generic shuffling. Both dominant *Cladocopium* were originally rare with relative abundances as low as 0.024%. This is the first record of adult corals switching dominant symbiont without bleaching.

## Introduction

Stable symbioses between scleractinian corals and dinoflagellate algae (family: Symbiodiniaceae) are key for coral reefs to thrive in tropical oligotrophic waters. More than 90% of a coral’s nutritional requirements are provided by its symbiotic algae^1^. Interruption of such symbioses may force corals to expel their associated algae, which results in coral whitening, a phenomenon best known as bleaching. Temporary bleaching may not kill corals immediately, but recurrent and prolonged bleaching can be fatal^2,3^. Rising sea surface temperatures (SSTs) are one of the major contributors to coral bleaching^3^. Tropical/subtropical SSTs have increased 0.25–0.75 °C since 1880 due to anthropogenic global warming^4^. Over the past three decades, three mass bleaching events have occurred on a global scale: 1997–1998, 2010, and 2015–17^4,5^. The intervals between bleaching events are decreasing and the duration of each event is lengthening. To survive, both corals and their symbiotic algae need to adapt to the rising SSTs.

Coral-associated Symbiodiniaceae currently comprises seven genera and several unnamed but genetically distinct lineages^6^. Two common genera in the Indo-Pacific—*Cladocopium* (formerly clade C) and *Durusdinium* (formerly clade D)—are the only Symbiodiniaceae detected in coral colonies from Taiwanese waters^7–10^. *Cladocopium* is the most diverse genus^6,11^, while members of *Durusdinium* are known to be resilient to hostile environments, e.g., high temperature or turbidity^2,6,9,10,12–27^.

In response to rising SSTs, corals and their algal symbionts use different strategies to acclimatize. In some cases, corals expel suboptimal algal species and host more optimal partners^28,29^. Some coral species host different algal symbionts when living in more hostile environments^15,23,30–32^, and some host multiple algal species and adjust the relative abundance of their symbionts, or shuffle between completely different symbionts when necessary^9,10,13,27,33–36^. Most studies have identified the dominant and codominant algal species in corals using conventional Sanger sequencing, restriction fragment length polymorphism (RFLP), quantitative polymerase chain reaction (qPCR), or temperature gradient gel electrophoresis (DGGE). However, these methods have limited resolution and very often fail to detect background species that might be ecologically important but extremely low in quantity (e.g., <5% relative abundance)^19,37^. High-throughput sequencing of the internal transcribed spacer 2 (ITS2) region is a promising approach to better understand intragenomic diversity and differentiate symbionts^38,39^. It can detect rare symbionts with relative abundances <1%. For example, using this approach, Boulotte *et al*.^40^ recorded rare symbionts in adult coral colonies and showed that symbiont switching occurred—one colony became dominated by one of the rare symbionts—after two consecutive bleaching events, a rare phenomenon in adult corals. In this study we applied the same approach to investigate Symbiodiniaceae dynamics in *Leptoria phrygia*, which is known to host *Cladocopium* or *Durusdinium*^8,23^, or both^8,41^, within the same colony.

We tagged and sampled 25 *L*. *phrygia* colonies—13 in Wanlitong (WLT) and 12 in the outlet of the third nuclear power plant (OL) in southern Taiwan—to investigate spatial and temporal variation in Symbiodiniaceae communities^41^. We collected five cores from each colony; each core was 10~20 cm apart longitudinally and was sampled from the top to the bottom of the colony. Our survey demonstrated that, within individual colonies, there were no correlations between sampling position and the Symbiodiniaceae community; there were, however, positive correlations between the relative abundance of *Durusdinium* and mean maximum temperature, and between the relative abundance of *Durusdinium* and temperature variability^41^. Moreover, *L*. *phrygia* colonies native to OL were dominated exclusively by *Durusdinium glynnii* (formerly D1), while those native to WLT were dominated by *Cladocopium* C3w or C21a, or co-dominated by *Cladocopium* sp. and *D*. *glynnii*^41^. WLT is a sea surface temperature (SST) steady site (SS) with an average summer SST of 29–30 °C and daily fluctuation of <3 °C^41^, while OL is a SST variable site (VS) with an average summer SST of 32–33 °C—2–3 °C higher than any adjacent reef sites^10^—and daily fluctuations up to 10 °C^42^. Seasonal variation was only observed in colonies co-dominated by both genera; however, despite changes in relative abundance, the dominant species in the colonies remained the same^41^. Background information acquired from this study suggested: (1) *L*. *phrygia* can only modify the relative abundance of its symbionts if multiple symbionts exist in the same colony, but dominant symbionts do not change over time. (2) *L*. *phrygia* copes with variable environments better by hosting *Durusdinium*, and only associates with *Cladocopium* in more stable environments.

The background above begs several questions. Which Symbiodiniaceae are present in individual *L*. *phrygia* colonies? Does Symbiodiniaceae composition within the same colony change through time? Can *L*. *phrygia* respond to a new habitat completely different from its native one and, if so, how long does it take to adapt? To answer these questions, we narrowed our sampling to 10 colonies, five each randomly selected from the SS and VS. We reciprocally transplanted those colonies and monitored the dynamics of the Symbiodiniaceae community in each for 12 months.

## Results

### Transplantation experiment and seawater temperature at the two sites

All deployed nubbins survived the entire experimental period, except the five transplanted Wanlitong (WLT) nubbins of colony V, which died before the first sampling time in July 2015. Therefore, WLT colony V was excluded from subsequent analyses.

The mean monthly temperatures for July 2015, March 2016, and August 2016 were 29.8 ± 0.4 °C, 26.6 ± 0.7 °C, 24.4 ± 0.8 °C, and 29.8 ± 0.5 °C, respectively, in WLT and 29.4 ± 1.2 °C, 27.7 ± 0.8 °C, 25.9 ± 1.0 °C, and 30.4 ± 1.1 °C, respectively, in the third nuclear power plant (OL). The Mann-Whitney-Wilcoxon test demonstrated that the mean temperatures were significantly different between two sites for all four months (p < 0.05). It is noteworthy that the mean temperatures in OL were unseasonably low in July 2015, possibly due to a super typhoon passing by southern Taiwan and reducing the mean summer temperature in OL.

### Sequencing and BLAST search results

**Figure 1:**
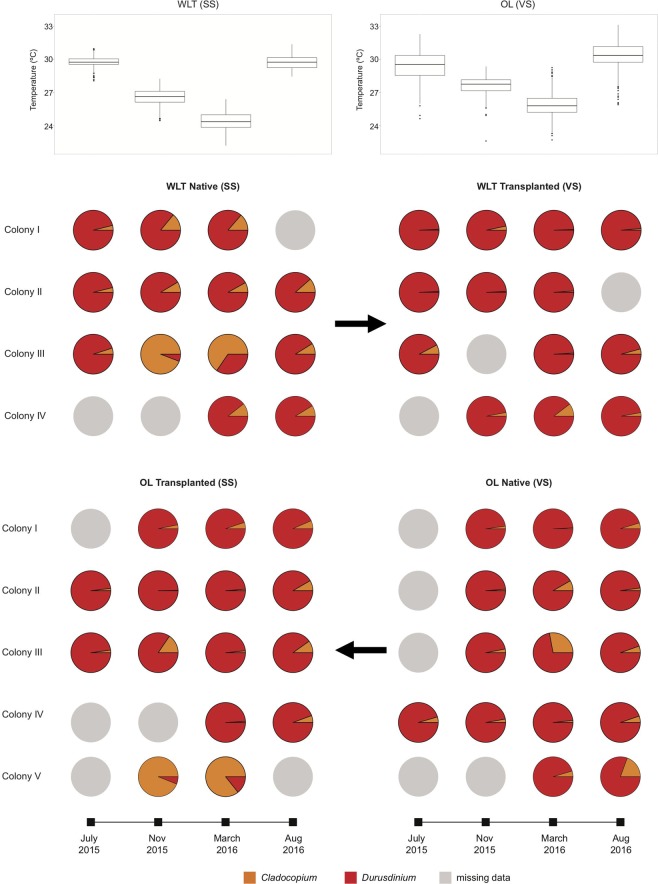
Temperature of four sampling months and seasonal dynamics of *Durusdinium* and *Cladocopium*.

The total number of reads in each library ranged from 4,527,909 to 6,123,654, and were reduced to 4,524,407 and 6,119,645 after quality trimming. The average read lengths were 274.1 to 284.5 bp (Table S1). Paired-read merging produced 9,795,746 assembled reads. After replicates were removed, 511,458 unique OTUs remained (56 nubbins in total). BLAST search against Symbiodiniaceae ITS2 references identified 80,236 sequences qualified for downstream analysis. Initial analysis revealed that 87.4% of those sequences belonged to *Durusdinium* and only 12.6% belonged to *Cladocopium*. Within individual nubbins, the first sampling in July 2015 showed that all nubbins, including native (control) and transplanted ones from both sites, exhibited an extremely high ratio of *Durusdinium* to *Cladocopium* (92~99%, Fig. 1).

Relative abundance analysis demonstrated that, in native WLT colonies, ratios of *Durusdinium* to *Cladocopium* were highest (≥90%) in summer (July 2015 and August 2016), when the SST was the highest (ca. 29.8 °C). In fall-winter (November 2015) and spring (March 2016), when the SST was cooler (ca. 26.6 and 24.4 °C, respectively), the *Durusdinium* to *Cladocopium* ratios decreased slightly in colonies I, II, and IV. In colony III, however, the *Durusdinium* to *Cladocopium* ratio decreased dramatically to as low as 6%, while the *Cladocopium* to *Durusdinium* ratio increased to 94%, becoming the dominant Symbiodiniaceae genus in November (Fig. 1). The *Cladocopium*-dominant stage lasted until March, but the *Cladocopium* to *Durusdinium* ratio decreased to 65%, then to 9% in August, at which time *Durusdinium* increased to 91% and regained dominance (Fig. 1). In OL, ratios of *Durusdinium* to *Cladocopium* were mostly >90% for every month, except in colonies III and V, which had a *Durusdinium* to *Cladocopium* ratio of 72% in March and 81% in August 2016 (Fig. 1). In transplanted WLT colonies, the *Durusdinium* to *Cladocopium* ratios were all>90%, without seasonal variations. In transplanted OL colonies, the *Durusdinium* to *Cladocopium* ratios remained high (>80%), without seasonal variation, except in colony V, which had no summer samples but did have a *Durusdinium* to *Cladocopium* ratio as low as 6 and 14% in November 2015 and March 2016, respectively.

The chi-squared tests indicated that the relative abundances of *Durusdinium* and *Cladocopium* were significantly (p < 0.05) or highly significantly (p < 0.001) different (Table 1) between native and transplanted samples in all WLT samples, regardless of the colony or season. In colonies I and II of the OL samples, the relative abundances of *Durusdinium* and *Cladocopium* between native and transplanted samples were not significantly different in November, but were in March and August. For colony III, the difference was highly statistically significant (p < 0.001) at all sampling times (Table 1). It was not different for colony IV, but was highly significantly different for colony V (Table 1).

**Table 1:** Chi-squared tests for the differences in relative abundances of *Durusdinium* and *Cladocopium* between native (control) and transplanted samples of each colony.

WLT	2015		2016		OL	2015		2016	
	July	November	March	August		July	November	March	August
colony I	<0.001 **	<0.001 **	<0.001 **	—	colony I	—	0.2042	<0.001 **	<0.05*
colony II	<0.001 **	<0.001 **	<0.001 **	—	colony II	—	0.0313	<0.001 **	<0.001 **
colony III	<0.001 **	—	<0.001 **	<0.001 **	colony III	—	<0.001 **	<0.001 **	<0.001 **
colony IV	—	—	<0.05*	<0.001 **	colony IV	—	—	0.1111	0.5787
colony V	—	—	—	—	colony V	—	—	<0.001 **	—

“—” means that a pair-wise test was not available due to missing samples; “*” denotes statistically significant and “**” denotes highly statistically significant.

### Composition and dynamics of dominant ITS2 sequences

**Figure 2:**
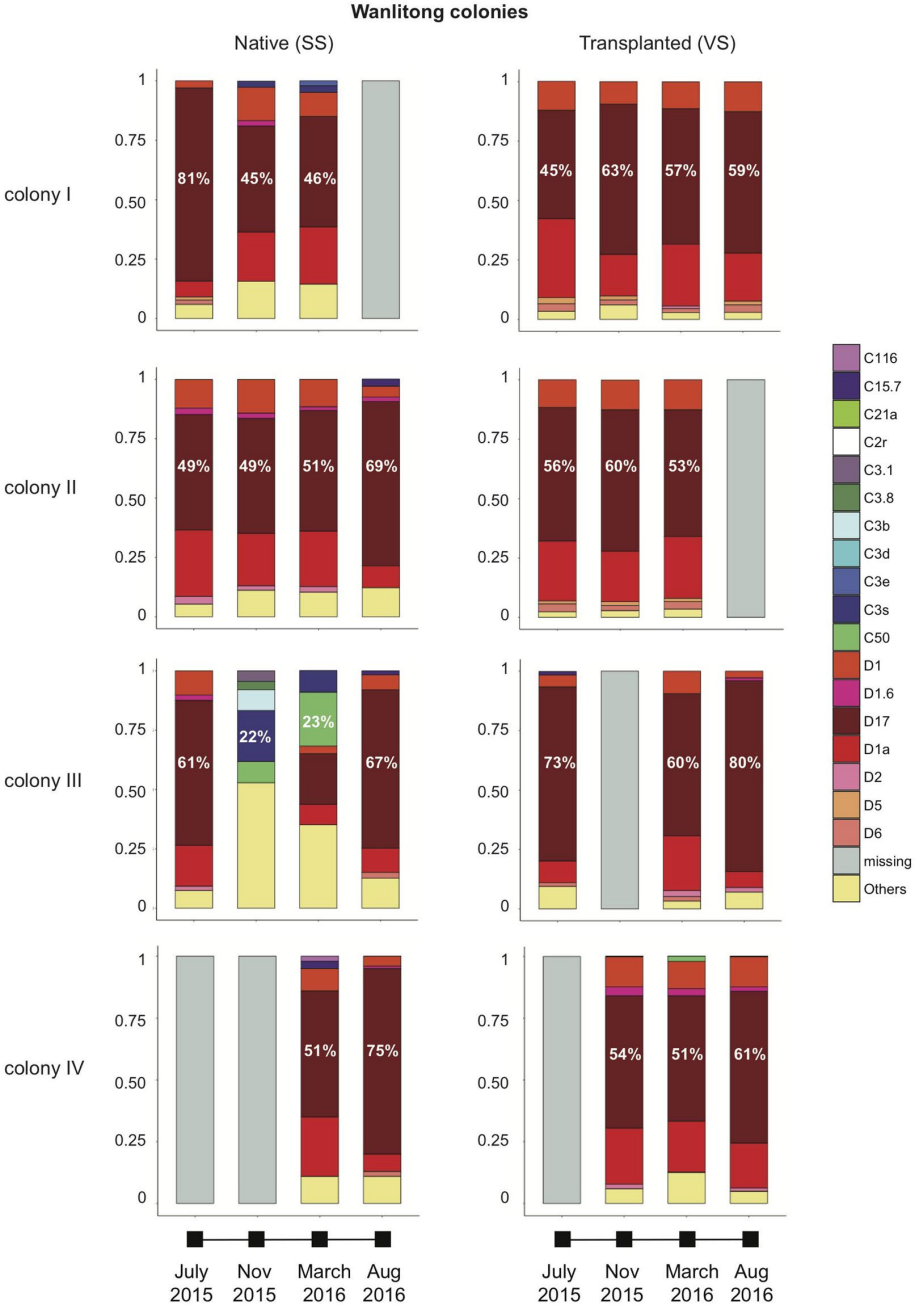
Top five symbionts in WLT colonies. Symbionts other than top five were pooled and defined as Others. Percentages of the most dominant symbionts are labeled.

**Figure 3:**
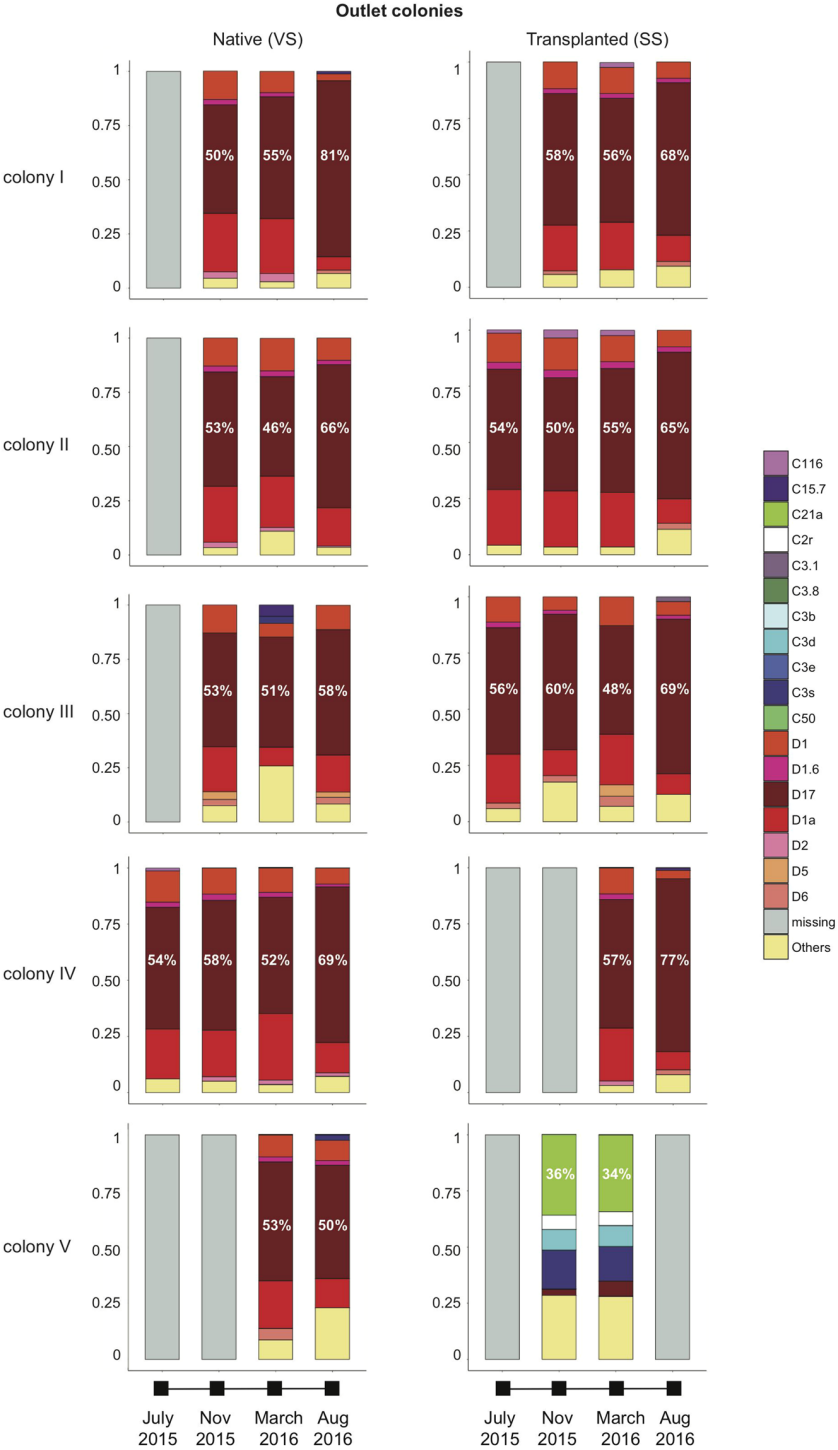
Top five symbionts in OL colonies. Symbionts other than top five were pooled and defined as Others. Percentages of the most dominant symbionts are labeled.

Symbiodiniaceae profiling demonstrated that both *Durusdinium* and *Cladocopium* were present within every individual *L*. *phrygia* nubbin. In most of the cases, the relative abundance of the top five taxa accounted for over 80% of the total ITS2 sequences within individual samples; therefore, only the relative abundances of the top five were listed, and the rest were pooled and classified as “Others.” Regardless of the site or season, *L*. *phrygia* colonies can be defined as having *Durusdinium*- and *Cladocopium*-dominant stages. During the *Durusdinium*-dominant stage, the relative abundance of the top three were always *Durusdinium* D17, *D*. *trenchii* (D1a), and *D*. *glynnii* (D1) (Figs. 2–3). Moreover, the relative abundance of the three occupied 65~95% of all Symbiodiniaceae in each colony.

**Figure 4:**
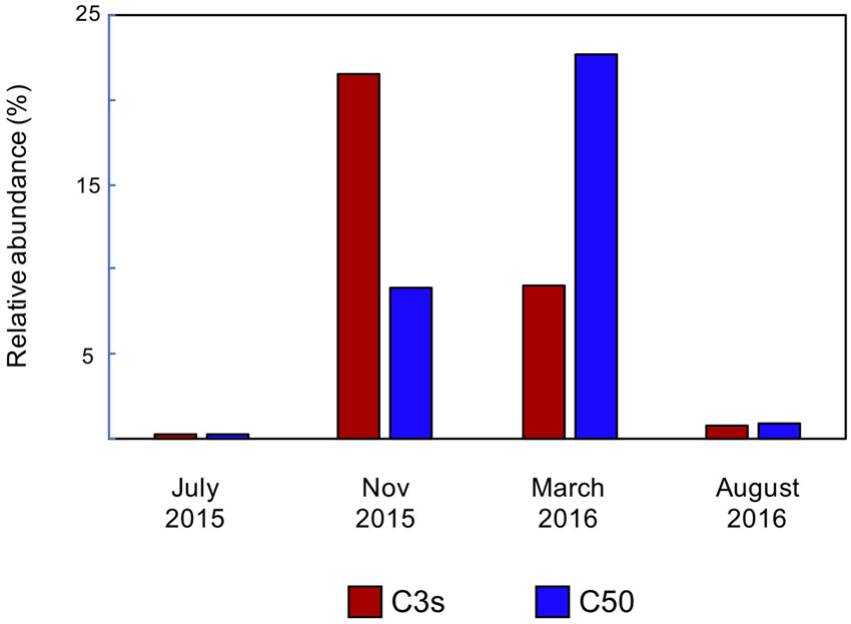
Switching in the most dominant *Cladocopium* in WLT colony III.

**Figure 5:**
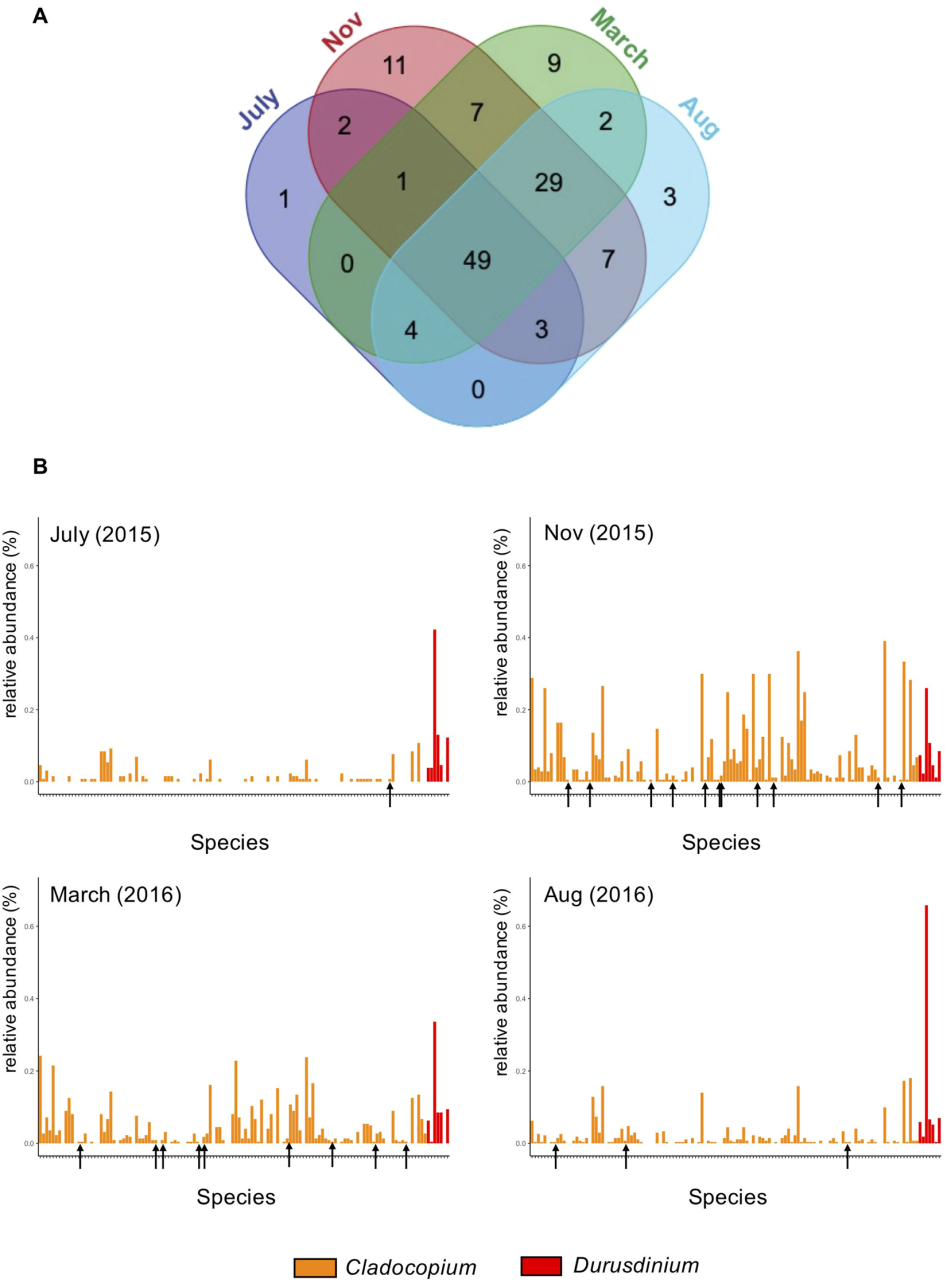
Analysis of rare symbionts. (**A**) Venn diagram of shared and unique symbionts among sampling months. (**B**) Distribution of rare symbionts in each month. X axis displays 128 other than the top five symbionts. Black arrows denote unique symbionts that only appeared in that sampling month.

During the *Cladocopium*-dominant stage, however, the relative abundances of the top three were more variable. For example, in WLT colony III, the top three most abundant taxa changed from *Cladocopium* C3s, C50, and C3b in November 2015 to C50, D17, and C3s in March 2016 (Fig. 2). In OL-transplanted colony V, the top three most abundant symbionts in November and March were C21a, C3s, and C3d (Fig. 3). Seasonal tracking of C3s and C50 in WLT colony III showed that the relative abundance of the two were as low as 0.24% in July, when the SST was the highest (ca. 29.8 °C) and the colony was dominated by *Durusdinium*. However, in November 2015, when the SST decreased to ca. 26.6 °C, the relative abundance of C3s increased to 21.5% and became the most abundant symbiont (Fig. 2). In March 2016, the highest relative abundance switched from C3s to C50 (which increased to 22.68%) when the SST further decreased to 24.4 °C. In August 2016, when the SST increased to >29 °C again, the relative abundance of both C3s and C50 decreased to <1% (Fig. 4) and the colony became dominated by *Durusdinium* again (Fig. 2). A combined analysis of all colonies from both sites searching for rare symbionts (relative abundances of the top five were excluded) also demonstrated an interesting trend: relative abundances of rare *Cladocopium* were low in July and August, when the SST was the highest. When the SST decreased in November, the relative abundance of rare *Cladocopium* increased (Fig. 5). However, when the SST further decreased in March, the relative abundance of *Cladocopium* also decreased. In addition, new *Cladocopium* ITS2 sequences appeared in each sampling month, showing peak diversities in November (11 total) and March (9 total). However, those sequences were transient, and none survived to the next sampling month (Fig. 5).

## Discussion

Our study demonstrated that ITS2 sequences in *L*. *phrygia* comprise few *Durusdinium* and numerous background *Cladocopium*. For the first sampling (July 2015), all colonies at both sites were particularly dominated by *Durusdinium* (relative abundance >90%). Our results concur with those of Carballo-Bolaños *et al*.^41^ in that colonies able to adjust their relative abundances of *Durusdinium* and *Cladocopium* mostly retained the same dominant symbiont through time; there were two exceptions: (1) native WLT colony III and transplanted third nuclear power plant (OL) colony V both shuffled from *Durusdinium*- to *Cladocopium-*dominated in November 2015 and March 2016, when the SST decreased (Fig. 1), and (2) native WLT colony III shuffled to become *Durusdinium*-dominant again when the SST increased again in August 2016 (Fig. 1). Instead of the *D*. *glynnii* and C3w or C21a recognized in Carballo-Bolaños *et al*.^41^, dominant symbionts in this study were D17 and C3s, C50, or C21a (Figs. 2–3).

Moreover, our Symbiodiniaceae profiling demonstrated that, for colonies dominated by *Durusdinium*, the top three most dominant symbionts were always D17, *D*. *trenchii*, and *D*. *glynnii*, and together they constituted more than 65% (majority >90%) of the Symbiodiniaceae. In contrast, *Cladocopium*-dominated colonies depended on the accumulation of different *Cladocopium*, each with a low relative abundance; even the most abundant taxon made up only ca. 21~22% (C3s and C50 in Fig. 2) or 34% (C21a in Fig. 3) of all Symbiodiniaceae. Most interestingly, in addition to dominant genus shuffling after the SST decreased in November (26.6 °C), we also recorded a switch in dominant *Cladocopium*—i.e., C3s to C50 in WLT colony III when the SST decreased two more degrees (24.4 °C) in March. Based on the results above, we can infer that *L*. *phrygia* has acclimatized to maintain a strong and stable association with three *Durusdinium*— D17, *D*. *trenchii*, and *D*. *glynnii*—but remained flexible enough to form a short-term association with *Cladocopium*.

Hosting a great quantity of *Durusdinium*, however, comes at a cost. *Durusdinium* is known as an opportunist that seizes the chance to become dominant in corals facing adverse conditions, and *Durusdinium* dominancy often has negative effects on coral health^26,43^. However, for corals living on the edge—e.g., the VS in our study—retaining considerable quantities of *Durusdinium* seems unavoidable. Our transplantation experiment validated the advantages of associating with *Durusdinium* when environmental conditions deteriorate: one colony died and the four surviving colonies all hosted *Durusdinium* exclusively (ca. 97% on average) after they were transplanted from the SS to VS. On the other hand, four of the five OL colonies remained *Durusdinium*-dominant, and the last one shuffled to become *Cladocopium*-dominant after transplantation from the VS (OL) to SS (WLT).

The evidence above suggests that the *Durusdinium* to *Cladocopium* ratio was so high (>92%) in all colonies in July due to the high SST combined with stress resulting from relocation, indicating aggravating ambient conditions. Although *L*. *phrygia* was able to shuffle to *Cladocopium* when the environment was less adverse—e.g., SST decreased—even switching to different dominant *Cladocopium*, the association with *Cladocopium* was only temporary. As soon as the SST increased again the next summer, the colony shuffled back to the same top three *Durusdinium*. Our results concur with another study on three coral species (*Pocillopora acuta*, *Acropora cytherea*, and *Porites rus*) that possess a unique Symbiodiniaceae signature: a suite of permanent Symbiodiniaceae genera in each colony^44^.

Previous studies show that corals do not take up algal symbionts indiscriminately^45^, nor do they change their dominant algal genus easily^46^. Moreover, before NGS techniques, the uptake of novel symbionts was only reported in corals at the juvenile stage, when the association was non-specific and flexible^47,48^. Even today, novel symbiont acquisition and switching in adult corals has only been reported in one case study—on *Pocillopora damicornis* after two consecutive bleaching events^40^. Our study is unique in that we monitored individual colonies and recorded the shuffling between *Durusdinium* and *Cladocopium*, as well as between dominant *Cladocopium* that switched within one particular colony over a period as short as 12 months without bleaching. This is the first record of a dominant symbiont switch in adult corals not triggered by bleaching.

However, although the uptake of novel symbionts by adult corals was reported in both Boulotte *et al*.^40^ (driven by bleaching) and our study (not driven by bleaching), whether or not the two cases truly constitute a switch is still equivocal. By definition, switching involves acquiring novel symbionts from the environment that later increase in abundance and dominate the colony^40,49^. The symbionts in these studies that switched actually existed in the colony before they were taken up, although their relative abundance was extremely low—0.03% in Boulotte *et al*.^40^ and 0.024% in our study. Nonetheless, we did detect new symbionts for each sampling month, but they never survived to the next sampling.

In conclusion, our study elucidated that *L*. *phrygia* has acclimatized to associate with three particular *Durusdinium* (D17, *D*. *trenchii*, and *D*. *glynnii*), but keeps numerous background *Cladocopium* in stock that may increase in abundance to dominate the colony when ambient conditions are less adverse. Sequences of new taxa are sporadically acquired from the environment, but they are transient and do not survive long enough to increase much in abundance. Consequently, *L*. *phrygia* may stand a chance of resisting recurrent and prolonged bleaching by solidly associating with *Durusdinium* supplemented with sporadic shuffling between *Durusdinium* and background *Cladocopium*. This coral is also resilient and recovers quickly from bleaching due to its swift responses to changing environments.

## Methods

### Sampling, temperature monitoring, DNA extraction, amplicon amplification, and sequencing

The study was conducted in Kenting National Park, southern Taiwan. We received a field permit (number: 1040008112) to conduct a reciprocal transplant experiment (spanning four seasons over 1 year) between Wanlitong (WLT) and the outlet of the third nuclear power plant (OL). The permit allowed us to sample 10 colonies from the two sites (WLT: 5 colonies, OL: 5 colonies) and collect a maximum of 10 subsamples from each colony, which was sufficient for our experimental design (80 samples in total for N = 5 per season/per site). Five *L*. *phrygia* colonies at 3–5 m deep from OL (21°55′53.7″N–120°44′42.7″E) and five colonies at 3–5 m deep from WLT (21°59′43.9″N–120°42′23.2″E) were haphazardly selected for subsample coring in March 2015. Sampled colonies were separated by at least 7–10 m to avoid sampling clones.

Procedures for coring and nubbin production were modified from Kao *et al*.^50^. Briefly, 10 cores (2.5 cm in diameter) were taken from each colony using a set of underwater drills. To reduce sampling effect, all cores were brought back to the laboratory in the National Museum of Marine Biology and Aquarium for processing and acclimatizing. In the laboratory, individual cores were attached to PVC tubes with epoxy (nubbins). Fifty nubbins collected from the same site were equally distributed on two fiberglass racks (25 nubbins each). On each rack, five nubbins from the same colony were arranged on the same row. Four racks (100 nubbins) were acclimatized for 96 hours and then returned to their native sites: two racks with WLT nubbins back to WLT and two racks with OL nubbins back to OL. The racks were fixed to the reefs at a depth of 3–5 m and allowed to acclimate for four weeks. In April 2015, one of the racks from each site was reciprocally transplanted to the other site, i.e., one WLT rack was transplanted to OL and vice versa. The other rack remained at the native site as the experimental control.

Data loggers (HOBO Pendant 64 K, USA) deployed at 3 m deep recorded ambient SST hourly from April 2015 to August 2016. Seasonal variation in the Symbiodiniaceae community compositions of native and transplanted nubbins were examined by collecting one nubbin per colony every three to four months. Nubbins were sampled in July 2015 (summer), November 2015 (fall-winter), March 2016 (spring), and August 2016 (summer). A total of 80 nubbins were collected from the racks by scuba diving, but some were lost during the sampling process. Consequently, fewer than 80 nubbins were brought back to the laboratory for molecular study. In the laboratory, a small piece of coral tissue was collected from each nubbin and fixed in 100% ethanol for DNA extraction using a modified high salt protocol^51^. Briefly, about 30 mg of coral tissue (~3–4 polyps) was fragmented and incubated at 55–60 °C overnight in a 200-μl lysis buffer mixture (recipes for 100 ml solution were: 1 M Tris-Boric 25 ml, 0.5 EDTA pH 8 10 ml, 20% SDS 10 ml, 5 M NaCl 2 ml, ddH_2_O 53 ml) and pronase E (10 μl at 10 mg/ml). To precipitate the DNA, 210 μl NaCl (7 M) was added to the lysis solution and mixed by inverting the tubes several times. The mixture was centrifuged at 10,000 × g for 30 min. The resulting supernatant was transferred into a new tube, mixed with 100% isopropanol (420 μl) for 5 min, and incubated at −20 °C for at least two hours. To clean the DNA, samples were centrifuged for 30 min at 16,000 × g and the supernatant was discarded. The DNA pellet was rinsed with 70% cold ethanol (150 μl) three times and dried in a laminar hood for 1–2 hr. A total volume of 150 μl preheated (65 °C) TE buffer (1×) was added to elute the DNA.

The primers ITSintfor2 (5′-GAATTGCAGAACTCCGTG -3′) and ITS2 (5′-GGGATCCATATGCTTAAGTTCAGCGGGT -3′) were used to amplify the ITS2 spacer along with partial ribosomal RNA genes (5.8 S and 28 S). Sixteen unique barcodes (6-bp) were attached to the 5’ end of the forward primer (ITSinfor2) for Illumina multiplex sequencing. Taq DNA polymerase RED (Ampliqon) was used for PCR amplification using the following conditions: initial denaturation at 95 °C for 5 min, followed by 35 cycles at 95 °C for 30 s, 53~55 °C for 30 s, 72 °C for 30 s, and a final extension at 72 °C for 10 min. The resulting amplicons were purified using the Gel/PCR DNA Purification Mini Kit (NovelGene, Taiwan) and measured with Nanodrop to determine the concentration and purity. A total of five tubes were prepared and delivered to Yourgene Bioscience, Co. (New Taipei City, Taiwan) for library construction and amplicon sequencing using Illumina MiSeq, 2 × 300 bp paired-end (MiSeq Reagent kit v3, Illumina Inc., CA, USA). Each tube contained 16 samples that could be distinguished by the unique barcode attached to the 5’ end of the forward primer.

### Data processing, ITS2 database establishment, and data analysis

Initial read trimming (error probability <0.05); barcode sorting; and adaptor, primer, and barcode removal were performed by Yourgene Bioscience, Co. (New Taipei City, Taiwan). Read lengths shorter than 35 bp and unpaired reads were removed from downstream analyses. Paired forward and reverse reads were further trimmed (PHRED quality score = 33, quality score threshold = 30) and then merged using PEAR v0.9.10^52^. For each sample, the merged reads were imported independently into QIIME^53^ for operational taxonomic unit (OTU) selection using the UCLUST method and a 97% similarity threshold. A previous study demonstrated that 97% within sample clustering collapses intragenomic sequences and only preserves sequences that are most likely interspecific^54^. The most abundant sequence variant in each 97% cluster was selected as the representative OTU. Only representative OTUs were used in downstream analyses. The identity of each representative OTU was determined by BLAST search against reference sequences from Arif *et al*.^38^ and Tong *et al*.^55^ with an e-value threshold of 10^−5^. The output with length coverage <250 bp or identity <97% were removed from further analyses. The relative abundances of *Cladocopium* and *Durusdinium* were calculated by the equation: total *Cladocopium* OTUs or total *Durusdinium* OTUs divided by total OTUs. All raw reads were deposited into GenBank and will be accessible once this manuscript is published (accession number PRJNA580156).

### Statistical analyses

Mean monthly temperature was calculated for each site, and the differences between the two sites over four sampling months were determined by non-parametric Mann-Whitney-Wilcoxon test. Differences in relative abundance between native and transplanted samples of each colony were evaluated by chi-squared tests. All statistical analyses were performed in R (version 3.4.2). Shared and unique symbionts across seasons were calculated through the Bioinformatics & Evolutionary Genomics online server (http://bioinformatics.psb.ugent.be/webtools/Venn/).

## Supplementary information

Table S1. Summary of MiSeq paired-end sequencing.
