# Supplementary material for: Leptoria phrygia in Southern Taiwan shuffles and switches symbionts to resist thermal-induced bleaching
Source: Sci Rep. 2020 May 8;10:7808. doi: 10.1038/s41598-020-64749-z (PMC7210888; doi:10.1038/s41598-020-64749-z)
Supplement: Supplementary file 1 — Table S1. Summary of MiSeq paired-end sequencing. [file 41598_2020_64749_MOESM1_ESM.pdf]

Leptoria phrygia in Southern Taiwan shuffles and switches symbionts to resist thermal-induced bleaching

Ya-Yi Huang[1#], Rodrigo Carballo-Bolaños[1, 2#], Chao-Yang Kuo[1], Shashank Keshavmurthy[1], Chaolun A. Chen[1,2,3,4*]

[1] Biodiversity Research Center, Academia Sinica, Taipei, Taiwan

[2] Biodiversity Program, Taiwan International Graduate Program, Academia Sinica; National Taiwan Normal University, Taipei, Taiwan

[3] Institute of Oceanography, National Taiwan University, Taipei, Taiwan

[4] Department of Life Science, Tung-Hai University, Taichung, Taiwan

# Co-first authors

*Corresponding author: Chaolun Allen Chen, E-mail address: cac@gate.sinica.edu.tw

Table S1. Summary of MiSeq paired-end sequencing.

	Library 1	Library 2	Library 3	Library 4	Library 5
Total reads	5,691,600	4,527,090	5,299,292	6,123,654	4,714,312
Read length	301	301	301	301	301
Total reads after QT	5,688,535	4,524,407	5,296,049	6,119,645	4,710,802
Average length after QT	279.2	284.5	283.1	277.1	274.1

QT: quality trimming (criteria: minimal length= 35 bp, error probability < 0.05)
